# Combination of metformin and double antibiotic paste for the regeneration of non-vital immature teeth: a preliminary randomized clinical study

**DOI:** 10.1186/s12903-023-03591-x

**Published:** 2023-11-11

**Authors:** Fereshte Sobhnamayan, Safoora Sahebi, Fariborz Moazami, Parastoo Malekzadeh, Soheila Hasani

**Affiliations:** 1 Department of Endodontics, Faculty of Dentistry, Shiraz University of Medical Sciences, Qasrdasht St., Shiraz, 71956-15878 Iran; 2 Department of Endodontics, Faculty of Dentistry, Lorestan University of Medical Sciences, Lorestan, Iran; 3 Department of Endodontics, Faculty of Dentistry, Yasuj University of Medical Sciences, Yasuj, Iran

**Keywords:** Disinfection, Double antibiotic paste, Immature teeth, Mesenchymal stem cells, Metformin, Non-vital, Root canal medicaments

## Abstract

**Background:**

The present study aimed to investigate whether incorporating metformin in double antibiotic paste (DAP) can promote the regeneration process of non-vital immature teeth.

**Methods:**

Out of 32 pediatric patients undergoing regenerative endodontic procedures (REPs), 6 cases with a follow-up period of less than 12 months or lack of documentation were excluded then the remaining 26 were analyzed. 15 cases received DAP, and 11 cases received a DAP + metformin as the intra-canal medicament, kept for an average of 23 days. During 18 months of follow-up, clinical and radiographic examinations were performed to evaluate the treatment outcomes based on the resolution of apical periodontitis, root development, and the occurrence of intracanal calcification. The chi-square test was used for the statistical analysis (*P* < 0.05).

**Results:**

All patients demonstrated resolution of apical periodontitis; however, complete apical closure was only seen in 50% of the patients. The rate of apical closure and root length was significantly higher in the DAP + metformin group (*P* = 0.047), although the two groups were not significantly different in terms of root width (*P* = 0.184). Canal obliteration was seen in 15% of cases, all of which were in the DAP group.

**Conclusions:**

According to the present findings, metformin could promote root development in the regeneration process when incorporated in DAP.

**Trial registration:**

This clinical trial was registered on the Iranian Registry of Clinical Trials (IRCT20200120046197N1) on 26.2.2021.

## Background

The aims of treatment in non-vital immature teeth are the resolution of disease, tooth retention, and, if possible, promotion of continued root development [1]. Their main management challenges are the thin dentinal walls, short roots, and fracture susceptibility in the long term [2]. As a regenerative biological treatment, REPs reinforce the root structure and facilitate the continuation of root development both from the width and the overall root length [3, 4]. Clinical and radiographic comparisons of regeneration and apexification have revealed that both treatments are 76 to 100% successful in facilitating further root development [1, 4, 5].

REPs owe their success mainly to the stem cells, signaling molecules, and a 3-dimensional physical scaffold, which empowers the empty canal to support the ingrowth of new tissues from the periapical area [6]. Periapical tissues in immature teeth have abundant blood vessels and contain stem cells, which can be instructed to self-renew and reconstruct the damaged part [7]. Biochemical factors are important signaling molecules that instruct dental papilla cells to achieve pulp regeneration [8].

In REPs, the necrotic root canals are disinfected to help the tissue proliferation into this space. The most commonly used intra-canal medicament in endodontic regeneration is triple antibiotic paste (TAP), which is a combination of metronidazole, ciprofloxacin, and minocycline. However, to avoid the discoloration caused by minocycline in the TAP, double antibiotic paste (DAP), a combination of metronidazole and ciprofloxacin, has been suggested and successfully used in endodontic regeneration [7]. Different antibiotics and growth factors have been added to synthetic or organic scaffolds to secure the success of REPs, and the results have been promising in both root development and healing of periapical lesions [9]. In a study by Nosrat et al. [10], five weeks of applying amoxicillin-clavulanate paste as an intra-canal medicament in immature teeth with acute abscesses resulted in complete periapical healing and formation of the root apex [10]. The treatment outcome can also be improved by scaffolding with platelet-rich plasma as it abundantly releases growth factors [11–13].

Metformin, an antidiabetic biguanide medication, is widely used to treat type II diabetes mellitus by controlling blood sugar levels [14]. This medication has been reported to have an osteogenic effect by promoting the differentiation of mesenchymal stem cells and pre-osteoblasts [15–18]. Wang et al. [19] found that adding metformin to a dental resin considerably enhanced the odontoblastic differentiation of dental papilla stem cells and increased their mineral synthesis, with potential applications to stimulate pulp cells for new dentin synthesis and bridge formation.

A clinical study showed that 1% metformin gel significantly improved the clinical and radiographic parameters in patients with chronic periodontitis [20]. Additionally, Sharma et al. [21] found that the combination of 1% metformin with platelet-rich fibrin in the treatment of grade II furcation defects significantly reduced the probing pocket depth and increased relative vertical and horizontal attachment levels.

Despite the approved ability of metformin for the differentiation of dental papilla cells into odontogenic cells, no study has ever investigated its application in the root canal system to help regeneration. The present study aimed to evaluate the effect of adding metformin to DAP on REPs in immature non-vital teeth. The null hypothesis was that adding metformin would not affect the success of regeneration in non-vital teeth.

## Methods

This preliminary randomized clinical trial adhered to the Consolidated Standards of Reporting Trials (CONSORT) guidelines. This study was a triple-blind (clinician, patients, and assessor) randomized clinical trial with 1:1 allocation. It was registered on the Iranian Registry of Clinical Trials (IRCT20200120046197N1), the full research protocol is available at: https://www.irct.ir. The allocation process was conducted by one of the contributing authors who did not participate in data analysis. The study received ethical approval from the Ethics Committee of Shiraz University of Medical Sciences (IR.SUMS.DENTAL.REC.1398.139). The participants were recruited from the pediatric patients referred to the Endodontic Clinic at the School of Dentistry of Shiraz University of Medical Sciences between 2017 and 2020. Informed consent was obtained from all participants or their guardians after clearly explaining the aims and probable risks and benefits of the study.

**Fig. 1:**
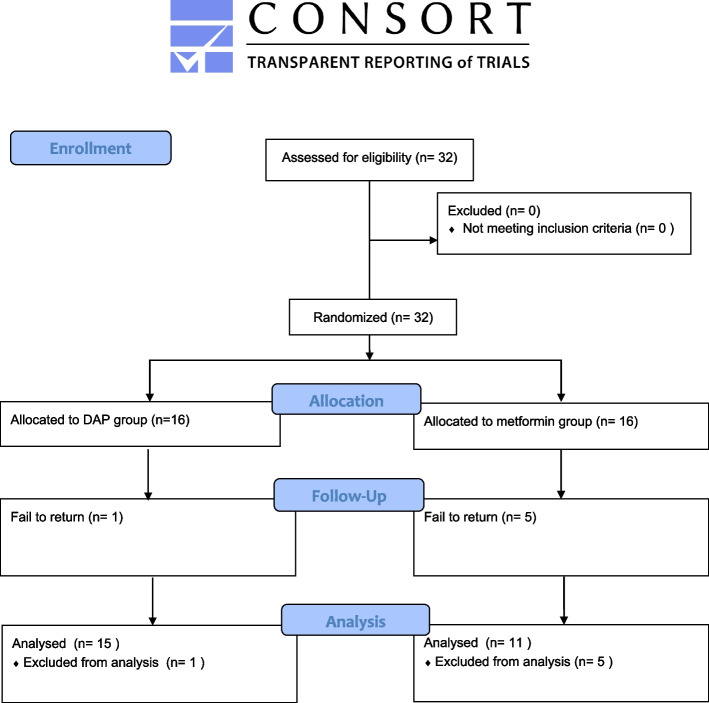
Flowchart of patients

 As a preliminary study, According to the previous studies [22, 23], 32 patients were included after assessing the inclusion criteria, and Block randomization was conducted with blocks of 4. The enrolled participants were 32 girls and boys aged 7 to 11 years, with permanent dentition, noncontributory medical history, and non-vital immature teeth with no response to vitality tests but restorable (with or without radiographic evidence of periapical lesions). Exclusion criteria were unrestorability, having teeth with mature roots, internal or external root resorption, root fractures, pre-endodontic treatments, severe periodontal diseases, poor oral hygiene, and uncooperativeness in follow-ups (Fig. 1).

### Interventions

The pre-treatment radiographs were taken with the standardized paralleling technique via the Rinn XCP alignment system (Rinn Corporation; Elgin, IL, USA). The periapical radiographs were digitized using a transparency scanner (HP Scanjet G3110; Hewlett-Packard Development Co, Palo Alto, CA, USA) for further comparisons.

Teeth were anesthetized by using local anesthesia with a vasoconstrictor (1.8 mg of xylopen 2%, Exir, Boroojerd, Iran). After rubber dam isolation, access cavities were prepared, and root canals were irrigated using 20 mL of 1.5% sodium hypochlorite (Cerkamed, Poland), minimally prepared, and dried with paper points. Then by using a #25 K-file (Mani, Tochighi, Japan), one group of the root canals (*n* = 16) was medicated with DAP (metronidazole [Parsdaru; Tehran, Iran], and ciprofloxacin [AminDaru; Tehran, Iran]) to a final concentration of 1 mg/mL, and the other group with (*n* = 16) DAP + metformin (Dr. Abidi Pharmaceuticals; Tehran, Iran) with the concentration of 1%. A sterile cotton pellet was applied and the access cavity was sealed with a temporary restoration (Cavisol; Golchai, Tehran, Iran) for 3 weeks.

The final visit was scheduled when the tooth was asymptomatic with no signs of discharge. In case of persistent infection, one or more visits were scheduled for further drainage and chemical disinfection. The average duration of keeping the medicament was 23 days, ranging from 21 to 28 days. In the final visit, after the application of anesthesia (mepivacaine 3% without vasoconstrictor (Exir Pharmaceutical, Tehran, Iran) and proper isolation, the temporary restoration and cotton pellet were removed. The canal was irrigated with 30 mL of ethylenediamine tetraacetic acid (EDTA) 17% (Morvabon; Tehran, Iran) followed by 10 mL sterile saline and dried with sterile paper points.

Intra-canal bleeding was induced with a #20 endodontic K-file (Mani, Tochighi, Japan) beyond the apical foramen. Upon the formation of a blood clot to the level of the cementoenamel junction, mineral trioxide aggregate (MTA, Angelus, Londrina PR, Brazil) was placed on the blood clot, and the teeth were restored with temporary restorations. The patients were referred to replace the restoration with permanent filling materials.

The patients were clinically and radiographically followed up for an average of 18 months. The follow-ups were first on a three-month basis (months 3 and 6), and thereafter, every 6 months. Each follow-up visit included clinical and radiographic examinations of the resolution of apical periodontitis. The pre-and post-treatment radiographic images were evaluated by two skilled independent examiners and classified as success and failure. The persistence of clinical signs and symptoms of periapical radiolucency was regarded as a failure. The root development was assessed based on the thickness and length of the root or apical closure. The data were statistically analyzed by using SPSS software (version 16, SPSS INC, Chicago, IL, USA). The inter-rater reliability was measured with Cohen’s kappa coefficient. The chi-square test was used to assess the effect of metformin incorporation into DAP on the root width, root length, apical closure, and periapical status. *P* values less than 0.5 were considered statistically significant.

## Results

Out of 32 patients undergoing REPs, 6 cases with a follow-up period of less than 12 months or lack of documentation were excluded then the remaining 26 were analyzed (Fig. 1). Table 1 displays the patients’ demographic data and relevant clinical parameters such as radiographic findings, preoperative symptoms, and periapical and pulpal diagnoses. The etiology of the pulpal disease was classified into trauma and caries and the mean age of the patients was 9.65 years. All the remaining 26 patients were diagnosed with pulp necrosis and different degrees of periapical presentation ranging from symptomatic apical periodontitis to chronic apical abscess with distinct periapical radiolucency.

**Table 1:** The patients’ demographic data and clinical findings

Case	Sex	Age	Tooth number	Etiology	Pulpal diagnosis	Periapical diagnosis	Symptoms	Periapical radiolucency	Soft tissue lesions
1	Male	10	19	Caries	Necrosis	AAP	Percussion (+)	-	None
2	Male	11	9	Trauma	Necrosis	AAA	Percussion (+)	+	Swelling
3	Male	9	8	Trauma	Necrosis	CAP	None	+	None
4	Female	7	8	Trauma	Necrosis	CAP	Percussion (+)	+	None
5	Male	11	30	Caries	Necrosis	CAA	None	+	Sinus tract
6	Male	11	8	Trauma	Necrosis	CAP	None	+	None
7	Female	12	22	Caries	Necrosis	AAP	Percussion (+)	+	None
8	Female	9	30	Caries	Necrosis	CAA	None	+	Sinus tract
9	Male	10	8	Trauma	Necrosis	AAA	Percussion (+)	+	Swelling
10	Male	8	8	Trauma	Necrosis	AAA	Percussion (+)	+	Swelling
11	Male	8	8	Trauma	Necrosis	Normal	None	-	None
12	Male	8	8	Trauma	Necrosis	Normal	None	-	None
13	Female	9	9	Trauma	Necrosis	CAA	None	+	Sinus tract
14	Female	9	10	Trauma	Necrosis	AAA	None	+	Swelling
15	Female	9	9	Trauma	Necrosis	AAA	Percussion (+)	+	Swelling
16	Male	8	8	Trauma	Necrosis	Normal	None	-	None
17	Male	10	10	Trauma	Necrosis	AAP	Percussion (+)	+	None
18	Female	12	22	Caries	Necrosis	CAA	None	+	Sinus tract
19	Female	10	21	Caries	Necrosis	CAP	None	+	None
20	Female	9	8	Trauma	Necrosis	AAP	Percussion (+)	+	None
21	Female	10	8	Trauma	Necrosis	AAP	Percussion (+)	-	None
22	Male	11	30	Caries	Necrosis	AAP	Percussion (+)	-	None
23	Male	10	9	Trauma	Necrosis	AAP	Percussion (+)	+	None
24	Male	9	9	Trauma	Necrosis	Normal	None	-	None
25	Male	9	8	Trauma	Necrosis	AAP	Percussion (+)	+	None
26	Male	12	21	Caries	Necrosis	CAA	None	+	Sinus tract

*AAP* Asymptomatic apical periodontitis, *AAA* Acute apical abscess, *CAP* Chronic apical periodontitis, *CAA* Chronic apical abscess

**Fig. 2:**
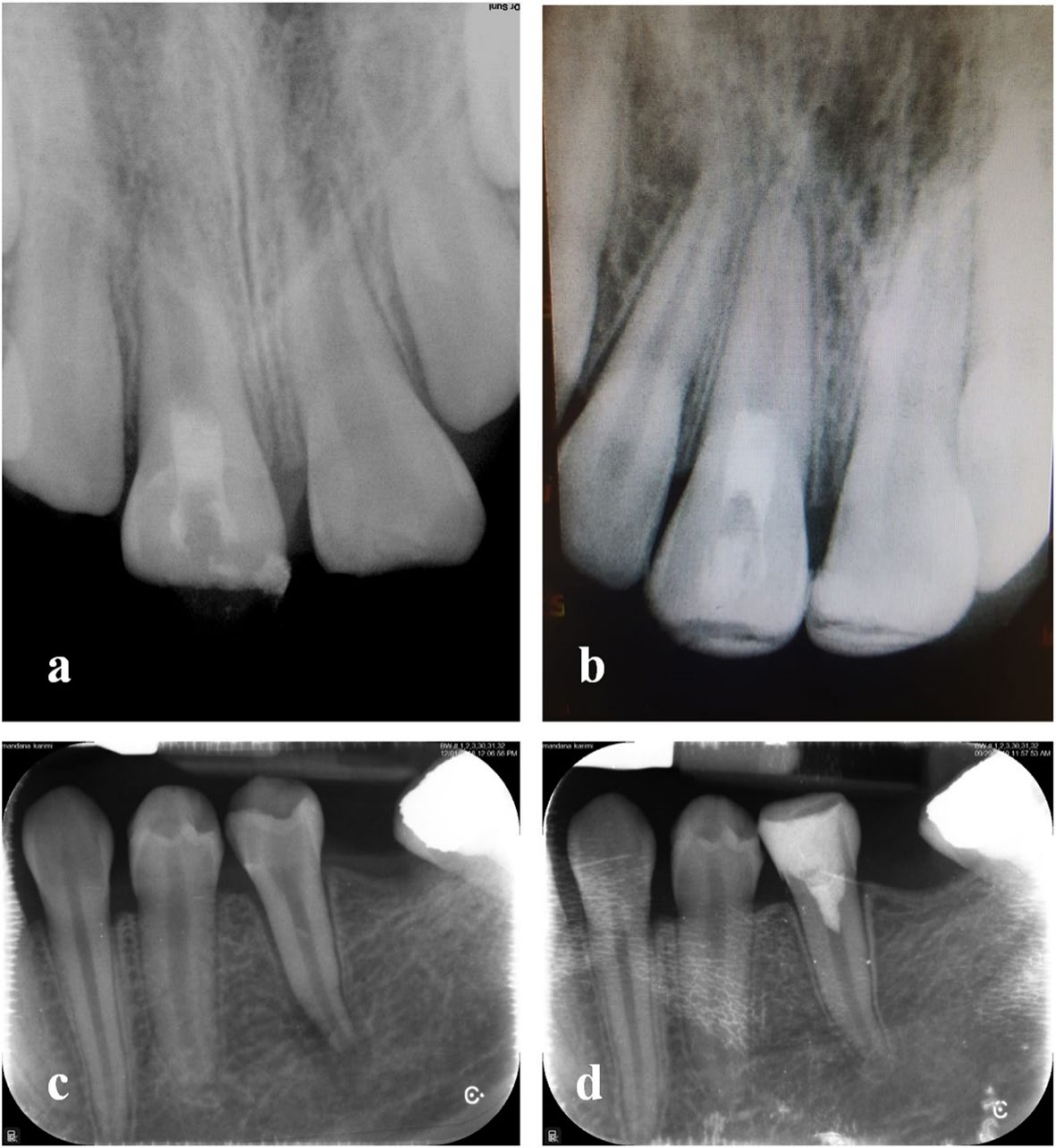
**a** Preoperative radiograph of tooth number 8. **b** At 24 month follow-up of tooth number 8. **c** Preoperative radiograph of tooth number 20. **d** At 18 month follow-up of tooth number 20

 There was an agreement between the two independent examiners on the radiographic assessment of the root development (Cohen’s kappa coefficient = 1.0). Radiographic and clinical examinations showed complete resolution of apical periodontitis and all cases were functional on follow-ups. Moreover, there was radiographic evidence of continued root development in terms of increased root length (*n* = 13) and thickness (*n* = 15). Complete apical closure was seen in 50% of cases (*n* = 13). Of 13 cases that were verified as negative, incomplete apical closure was seen in 27% of cases (*n* = 7), and only 23% showed no signs of apical closure (*n* = 6) (Fig. 2). The rate of apical closure and root length was significantly higher in the DAP + metformin group (*P* < 0.05); however, the two groups were not significantly different in terms of root width (*P* = 0.184). Canal obliteration was seen in 15% of cases (*n* = 4) who were all among those treated with DAP per se (Table 2).

**Table 2:** Statistical comparison of variables between the study groups

Variables	-/+	DAP (*n* = 15)	DAP + Metformin (*n* = 11)	*P* ^*^
Root width	-	8 (53.3%)	3 (27.3%)	0.184
	+	7 (46.7%)	8 (72.7%)	
Root length	-	10 (66.7%)	3 (27.3%)	0.047
	+	5 (33.3%)	8 (72.7%)	
Apical closure	-	10 (66.7%)	3 (27.3%)	0.047
	+	5 (33.3%)	8 (72.7%)	
Periapical status	-	2 (13.3%)	1 (9.1%)	0.738
	+	13 (86.7%)	10 (90.9%)	

*Pearson’s chi-square

## Discussion

The null hypothesis was rejected since the present results showed that adding metformin to DAP significantly increased the root height and the rate of apical closure compared to the DAP per se. All cases in both groups showed periapical healing and 77% of cases showed signs of apical closure; 50% complete apical closure and 22% incomplete apical closure.

REPs stand among one of the best strategies to treat immature permanent teeth with pulp necrosis [4, 24–26]. Song et al. [26] compared TAP, DAP, and Ca(OH)_2_ as intracanal medicaments to trigger regeneration. They observed continued root development in 23 out of 29 patients and complete apical closure in 27% of patients. Likewise, Chen et al. [27] used calcium hydroxide as an intra-canal medicament and reported continued root development in 15 of the total 20 studied cases. Petrino et al. [28] evaluated a case series of six immature necrotic teeth and observed continued root development in three cases, only two of which showed a positive response to the vitality test. All the above-mentioned studies were in agreement with the current study. The minor differences in the root development rate can be attributed to multiple factors such as the type of intracanal medicament and time of application, follow-up period, type of studied teeth, and degree of root development.

In contrast with the present study, Ding et al. [29] reported complete root development only in 25% of regenerated teeth. Such a difference may be due to the duration of applying the intra-canal medicament (one week versus three weeks) and strict criteria for success. They considered complete apical closure as a success instead of continued root development as in the present and other studies.

The present study showed complete clinical and radiographic resolution of apical periodontitis in all cases. Palma et al. [30] have demonstrated radiographic resolution of apical periodontitis and histologic evidence of survival of apical papilla stem cells in a hypoxic and infected environment, aligning with current research. Regenerative endodontic treatments have been shown to be successful in clinical studies [1, 31]. However, a systematic review conducted by Meschi et al. has concluded that there is currently insufficient evidence to support the effectiveness of REPS in treating apical periodontitis [32].

Various factors can affect REPs outcomes, for instance, tooth type and etiology. Lin et al. [31] reported the impact of etiology on treatment outcomes, in addition to apical healing and resolution of symptoms. Dens evagination cases have better prognosis and root development than trauma cases. In contrast, Jiang et al. [33] evaluated the regeneration outcomes in different tooth types with various etiologies of tooth necrosis. They reported an increase in root length and thickness, with a high success rate, regardless of the etiology of tooth necrosis or tooth type.

Meanwhile, radiographic evidence of pulp canal obliteration or hard tissue deposition was observed in 15% of cases in the present study (*n* = 4), which was lower than that in Chen et al.‘s (30%) [27] and Song et al.‘s studies (72%) [26]. Moreover, Song [26] documented pulp canal obliteration in 76.9% of cases medicated with calcium hydroxide, while this amount decreased to 46% in cases treated with TAP or DAP. Seemingly, using calcium hydroxide as intra-canal medicament would increase the risk of calcification in root canal space. Chen [27] suggested intra-canal calcification as a complication of internal replacement resorption, which was more probable in cases treated with calcium hydroxide.

Hard tissue deposition in the canal wall was also reported in other studies [25, 28, 29, 34]. The nature of the hard tissue in human studies is speculative as no histological studies are available. Animal studies describe these hard tissues as osteoid or cementoid tissues [35–38]. However, the mechanism of canal obliteration in regenerated immature permanent teeth is not clear; nor is the clinical and histopathological outcome of pulp canal calcification in regenerated teeth. The overall rate of canal obliteration in this study was similar to other studies; however, interestingly, all obliterated canals were those treated with DAP per se. Some studies reported the favorable effects of metformin on odontoblastic differentiation of mesenchymal stem cells and enhanced mineral synthesis and tissue regenerative properties [19, 39]. Consistent with these earlier findings, our study observed notable improvements in root regeneration and development within the metformin-treated group. Furthermore, the absence of canal obliteration observed in the metformin group in the present study warrants further investigations for a comprehensive understanding of its effects. Metformin is widely used for diabetes as a safe, non-toxic, and well-tolerated medication. In several attempts to investigate the effect of metformin on cell proliferation and mineralization, different concentrations of it have been assessed to find the most efficient and least cytotoxic concentration for dental papilla cells [16, 17, 40, 41]. Qin et al. [40] detected that metformin in concentrations below 50 µmol/L (10, 20, and 50) would not affect dental papilla cell viability. They also found that metformin significantly activated the adenosine monophosphate-activated protein kinase (AMPK) pathway in a dose-dependent manner, stimulated alkaline phosphatase activity, enhanced mineralized nodule formation, and increased the expression of odontoblastic markers including dentin sialoprotein, dentin matrix protein1, runt-related transcription factor 2, and osteocalcin.

Another study showed that the use of 200 µmol/L of metformin in the root canals of 20 rat teeth with periapical lesions diminished the size of periapical lesions through inhibition of hypoxia-enhanced mitochondrial superoxide production in osteoblast [42]. Liu et al. [43] injected 40 mg/kg of metformin into the rats with periapical lesions and found that systemic use of metformin inhibited the periapical lesions by reducing the osteoclast and bone resorption area. The number of RANKL-positive and tartrate-resistant acid phosphatase (TRAP)-positive cells in metformin-treated cases decreased after 14 days; whereas, the number of OPG-positive cells increased after 28 days.

Pradeep et al. [44] used 0.5, 1, and 1.5% metformin gel as a local drug delivery system in addition to scaling and root planning in patients with chronic periodontitis. They noted that local delivery of metformin into the periodontal pocket greatly decreased the probing depth and reduced the intrabony defects. In another try to treat intrabony defects in patients with chronic periodontitis, Pradeep et al. [39] combined 1% metformin gel with platelet-rich fibrin and the results were more promising than using metformin or PRF per se, as metformin per se did not show a sustained release into the area and diluted immediately. In the present study, to surmount the rapid dilution of metformin, DAP was used as a scaffold to act as both an antibacterial paste and to confine metformin to the root canal system. Presumably, the slow release of metformin could encourage the expression of odontoblastic markers and stimulate alkaline phosphatase activity [8], although the mechanism is still unknown.

One of the primary strengths of the present study is the randomized clinical trial design. Our study addresses an important clinical question in the field of regenerative endodontics. We acknowledge that one of the limitations of our study is the relatively small sample size. In addition, we recognize that our sample size calculation may not have been fully accounted for. Additionally, the incorporation of a wide spectrum of periapical conditions and variables within our study is recognized as a limitation due to clinical intricacies. Given the limitations of our current study, further histopathologic and clinical studies with more samples and longer follow-up periods are suggested to investigate the influence of certain factors, such as periapical conditions, on the success of treatment and prove the desired effects of metformin in regeneration.

## Conclusions

According to the results of the present study, the combination of metformin with DAP significantly promotes root development in comparison to the usage of DAP per se in regeneration. Hence, it may be deemed a potential adjunct to the double antibiotic paste in such treatment approaches.

## Data Availability

The datasets used and analyzed during the current study are available from the corresponding author upon reasonable request.

## Abbreviations

DAP: Double antibiotic paste
TAP: Triple antibiotic paste
